# Interferon-Based Anti-Viral Therapy for Hepatitis C Virus Infection after Renal Transplantation: An Updated Meta-Analysis

**DOI:** 10.1371/journal.pone.0090611

**Published:** 2014-04-03

**Authors:** Fang Wei, Junying Liu, Fen Liu, Huaidong Hu, Hong Ren, Peng Hu

**Affiliations:** 1 Department of infectious Disease, Institute for Viral hepatitis, Key Laboratory of Molecular Biology for infectious disease, The second Affiliated Hospital of Chongqing Medical University, Chongqing, PR China; 2 Department of Gastroenterology, The Central hospital of Zhoukou, Henan Province, China; Temple University School of Medicine, United States of America

## Abstract

**Background:**

Hepatitis C virus (HCV) infection is highly prevalent in renal transplant (RT) recipients. Currently, interferon-based (IFN-based) antiviral therapies are the standard approach to control HCV infection. In a post-transplantation setting, however, IFN-based therapies appear to have limited efficacy and their use remains controversial. The present study aimed to evaluate the efficacy and safety of IFN-based therapies for HCV infection post RT.

**Methods:**

We searched Pubmed, Embase, Web of Knowledge, and The Cochrane Library (1997–2013) for clinical trials in which transplant patients were given Interferon (IFN), pegylated interferon (PEG), interferon plus ribavirin (IFN–RIB), or pegylated interferon plus ribavirin (PEG–RIB). The Sustained Virological Response (SVR) and/or drop-out rates were the primary outcomes. Summary estimates were calculated using the random-effects model of DerSimonian and Laird, with heterogeneity and sensitivity analysis.

**Results:**

We identified 12 clinical trials (140 patients in total). The summary estimate for SVR rate, drop-out rate and graft rejection rate was 26.6% (95%CI, 15.0–38.1%), 21.1% (95% CI, 10.9–31.2%) and 4% (95%CI: 0.8%–7.1%), respectively. The overall SVR rate in PEG-based and standard IFN-based therapy was 40.6% (24/59) and 20.9% (17/81), respectively. The most frequent side-effect requiring discontinuation of treatment was graft dysfunction (14 cases, 45.1%). Meta-regression analysis showed the covariates included contribute to the heterogeneity in the SVR logit rate, but not in the drop-out logit rate. The sensitivity analyses by the random model yielded very similar results to the fixed-effects model.

**Conclusions:**

IFN-based therapy for HCV infection post RT has poor efficacy and limited safety. PEG-based therapy is a more effective approach for treating HCV infection post-RT than standard IFN-based therapy. Future research is required to develop novel strategies to improve therapeutic efficacy and tolerability, and reduce the liver-related morbidity and mortality in this important patient population.

## Introduction

Hepatitis C virus (HCV) infection is a significant public health problem, with an estimated 170 million people infected and three to four million new cases per year [1], [2]. HCV infection remains highly prevalent in patients with end-stage renal disease (ESRD) who undergo planned hemodialysis and renal transplantation [3]–[7]. Renal transplant (RT) recipients have a HCV infection rate of 5–15% in the developed countries, with substantially higher rates reported in the developing world [8], [9].

The immunosuppressed state of RT recipients dramatically increases the risk of HCV infection and accelerated disease progression. This condition can lead to severe HCV-related liver damage such as cirrhosis, fibrosing cholestatic hepatitis or liver failure. The risk of liver failure in particular is a major concern, as this condition is the fourth leading cause of mortality (8–28%) in long term survivors after RT [6], [10]. Furthermore, HCV also negatively impacts renal graft survival [11], [12]. Indeed, current evidence suggests that the long-term graft and patient survival rates of HCV-positive RT recipients were significantly lower than that of HCV-negative patients [13]–[15]. Thus, prevention and management of HCV infection is a critical factor in RT therapy.

IFN-based therapy is the primary treatment for HCV-related liver disease. However, in the renal transplant setting, the use of IFN therapy has produced unsatisfactory results. Not only are these therapies less effective, but they are also associated with increased risks of acute renal insufficiency and graft rejection [16], [17]. So, physicians managing RT recipients must balance the benefits of reducing HCV infection and subsequent hepatic disease with the complications from antiviral therapy.

The serious complications of HCV infection post-RT have led many researchers around the world to investigate the use of IFN-based antiviral therapy (immunotherapy or combination treatment) to attenuate the aggressive course of HCV infection post-RT. In 2006, a meta-analysis performed by Fabrizi et al [18] had evaluated the efficacy and safety of IFN/IFN-RIB therapy in this patients. However, this study did not include reports of PEG-based (PEG/PEG-RIB) therapies and only used various forms of the conventional IFN doses. Furthermore, Most of the included studies had small sample sizes and the meta-analysis did not include large randomized controlled trials, so the accuracy of these findings remains uncertain.

Currently, most antiviral strategies post-RT employs monotherapies (i.e. IFN/RIB/Amantadine/PEG) [19]–[28]. However, there are some case reports that describe successful treatment of chronic HCV infection in RT recipients using combination therapies (i.e. IFN-RIB/PEG-RIB) [29]–[32]. In particular, PEG-based therapies appear to have fewer side effects, better antiviral efficacy, and more rapidly viral clearance than the standard IFN therapy in most patients [16]. Since earlier meta-analyses did not include PEG-based therapy or combination therapy, an updated meta-analysis is necessary to evaluate IFN-based therapy more appropriately in post-RT patients.

The overall benefits and best strategies for treating HCV infection post-RTwith IFN therapy remain poorly understood. To evaluate the safety and efficacy of IFN-based therapies properly, we carried out a systematic review and an updated meta-analysis of the published clinical trials using of IFN-based monotherapies and combination therapies (IFN or PEG alone or IFN–RIB or PEG–RIB) of HCV infection post-RT. These findings should help determine the optimal treatment strategy for managing HCV in RT recipients.

## Materials and Methods

### Search strategy

We performed a comprehensive search of the published literature for controlled and observational studies regarding the efficacy of IFN-based therapy (IFN or PEG alone or IFN–RIB or PEG–RIB) for HCV infection post-RT. Studies from January 1997 through April 2013 were pulled from Pubmed, Embase, Web of Knowledge, and the Cochrane Library, using key words “HCV,”“interferon,” “renal transplant,” and their synonyms. The search was restricted using the terms “humans” and “English”; we obtained studies (controlled or non-controlled, randomized or non-randomized) published in full-text or in abstract form for all potentially relevant trials, and the reference list from retrieved documents were also searched to identify additional relevant studies.

### Study selection criteria

All retrieved citations were imported into Endnote X4.0.2 reference management software to remove duplicate reports. All potentially eligible full-text articles and abstracts were independently reviewed by two separate reviewers for relevance, inclusion in the meta-analysis, and data extraction using a standardized data collection form. Disagreements between reviewers were resolved with the assistance of an arbiter.

Given the heterogeneity in the published literature, strict inclusion and exclusion criteria were developed to capture all relevant literature, while excluding poorly conducted studies and limiting heterogeneity. If the same patients in different studies were reported two or more studies in controlled and non-controlled form, we included only the studies that reported the complete and adequate data that we needed.

The following inclusion criteria were used to select studies for meta-analysis: i) studies published as peer-reviewed articles; ii) study population must be renal transplant with HCV infection (positive for anti-HCV and/or HCV-RNA and/or biopsy proven) treated with IFN-based scheme (IFN or PEG alone or IFN–RIB or PEG–RIB) and reported the results of the treatment; iii) studies used the sustained virological response (SVR) and/or drop-out rate as a clinical end-point. Review articles, conference abstracts, interim reports of ongoing studies, case reports were excluded from the meta-analysis. In addition, we excluded studies that included patients co-infected with human immunodeficiency virus (HIV) and/or hepatitis B virus (HBV), patients undergoing multiple organ transplantation, clinical trials concerning patients on maintenance dialysis, and studies with inadequate response or treatment data.

### Data extraction and outcomes

Intention-to-treat methods were used to extract response rates for all patients in eligible studies. While patients without end-point data were excluded from our analysis. The primary outcome measure in this meta-analysis was SVR rate, a measure of efficacy, which was defined as HCV viraemia (HCV RNA in the blood) undetectable at least six months after cessation of treatment. The secondary outcome measure was Drop-out rate, a measure of tolerability, which was defined as the frequency of patients who stopped treatment due of side-effects.

Additional outcome measures included biochemical response, defined as normalization of serum alanine aminotransferase (ALT) at the end of treatment (ETBR) and at least 6 months of follow-up (EFBR). Virological response at the end of treatment (ETVR) and Rejection rate (the proportion of patients who experienced graft rejection) were also measured. In addition, we measured compliance among treatment groups (completion of full duration at original drug doses defined as A; completion of full duration but at reduced drug doses defined as B; premature termination of treatment defined as C).

### Statistical analysis methods

The response rate according to the intention-to-treat method was calculated by the data abstractor. Pooled quantitative summary estimates of the pre-defined outcome rates across individual studies were generated using the random-effects model of DerSimonian and Laird [33]. Unlike a simple arithmetic average, this estimate represents a weighted average of results from individual studies based on study size. The Q-test for heterogeneity was performed for each outcome measurement; a value of <0.10 was considered indicative of statistically significant heterogeneity [34]. The I squared (I^2^) value was calculate to assess the consistency of effects across studies [35]. Since the majority of studies in HCV infection post-RT utilized a non-controlled and non-randomized design, we performed the pooled quantitative analysis with consideration for the biases that may result from a lack of randomization [36]. We analyzed five stratifying variables (The SVR and Drop-out rate in Asian countries, in cohort studies, in patients treated with IFN-alone, IFN-RIB, and PEG-RIB).

To explore the potential effect of patients or trial characteristics on the summary estimates, a meta-regression analysis was performed [37]. The dependent variable was the observed logit event rate from each trial for the outcome of interest. Weights were assigned based on the estimated variance of logit event rate. The residual between-trial variance was estimated by a Restricted Maximum Likelihood Method (REML) using an iterative procedure [37]. The following covariates were included in the meta-regression analysis: Age, male percentage, reference year, rate of cirrhosis, donor source (cadaveric/living), duration of post-RT time before antiviral therapy, duration of antiviral therapy, and IFN dose. A sensitivity analysis using a random-effects model was also performed to assess the consistency of results. Publication bias was assessed by the Begg and Mazumadar adjusted rank-correlation test and by a regression asymmetry test for publication bias [38]. Every estimate was given with its 95% confidence interval (95% CI), with an alpha risk of 0.05. All the statistical analyses were performed using Stata 12.0 (Stata Corporation, College Station, TX, USA).

## Results

### Search results

**Figure 1:**
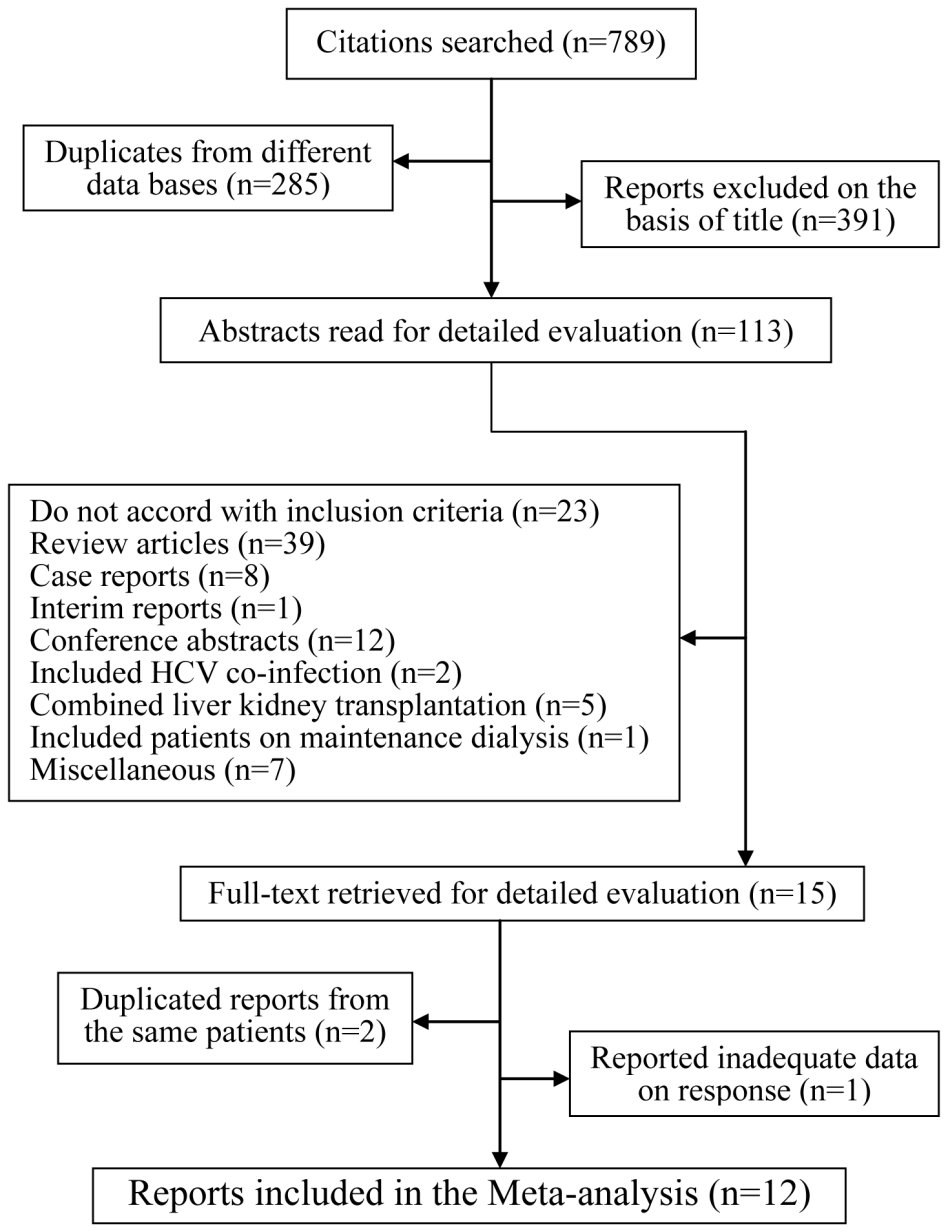
Map of the literature search and selection process.

According to the search strategy (Figure 1), 789 relevant reports were identified within the searched databases, of which 285 were redundant documents between two or more databases. An additional 391 reports were excluded on the basis of title, resulting in 113 eligible trials. Of these, 39 reports were review articles; eight were case reports [19]–[23], [29], [30], [39]; one was an interim report [40]; 12 were conference abstracts [24]–[28], [31], [32], [41]–[45]; 2 included HCV co-infected with HBV [46], [47]; five were combined liver kidney transplantation [48]–[52] and one included patients on maintenance dialysis [53] at the same time, and seven articles contained confounding factors [54]–[60]. After these exclusions, 12 reports met our eligibility criteria and were included in the meta-analysis [61]–[72].

### Patient characteristics

In Table 1, the lists of studies were analyzed. Seen from the chart, a total of 12 reports, describing a total of 140 patients were included. All of the reports were published in English and conducted between 1997 and 2013. Among them, 11 were conducted as cohort studies, only one used a controlled design approach, although none were randomized, controlled trials (RCTs). Many of the studies were performed in Asian countries (n = 7; 58%). The mean age of the patients ranged from 37 to 52.2 years and the men represented 59.4% to 100% of the study population. The cadaveric source of the donor was recorded in six of the 12 studies (50%). With regard to the viral characteristics, the genotype was reported in eight of the studies indicating that genotype-1 HCV infection predominated in these reports. Also, a liver biopsy was performed in most of the studies (9/12; 75%) suggesting that the frequency of cirrhosis was very low in these patient groups.

**Table 1:** Characteristics of studies of IFN-based therapy for HCV infection post-RT.

Author	Study Design	Reference Year	Total(n) Male (%)	Age (Year)	Cadaveric donor source	Geno-type 1	Geno-type 2	Cirrhosis %(n/T)
YasumuraT.et al [62]	Co,R	1997	6;100% M	37±5	NA	67.7% (4/6)	33.3%(2/6)	0
Izopet J.et al [61]	Co,P	1997	15;68% M	49 (29–65)	100%	86.7% (13/15)	13.3% (2/15)	13.3% (2/15)
Durlik M.et al [63]	Co,P	1998	11;73% M	38 (20–63)	100%	NA	NA	0
Hanafusa T.et al [64]	Co,P	1998	10; NA M	NA	NA	90%(9/10)	10% (1/10)	0
Tokumoto T.et al [65]	Co,P	1998	6; 83% M	46.8±6.6	67.7%	50%(3/6)	50% (3/6)	0
Baid S.et al [66]	Co,R	2003	12;75% M	48 (30–75)	83.3%	NA	NA	0
Tang S.et al [67]	Co,P	2003	4,100% M	45.8±6.8	100%	50%(2/4)	25% (2/4)	0
Shu K.H.et al [68]	Co,P	2004	11;73% M	42.4±13.1	100%	67.7%(6/9)	33.3% (3/9)	NA
Sharma R.K.et al [69]	CCT	2006	6; NA M	38.7±11.2	NA	NA	NA	NA
Pageaux G.P.et al [70]	Co,R	2009	8;100% M	52.2±5.6	NA	25% (2/8)	50% (4/8)	NA
Aljumah A.A.et al [71]	Co,R	2012	19;68% M	39.9±12.6	NA	NA	NA	0
Sanai F.M.et al [72]	Co,P	2013	32;59% M	46.0±12.4	NA	62.5%(20/32)	0	0

In Table 2, the specific treatment schedules are shown, which included the time of IFN-based treatment initiation after renal transplantation, the doses of IFN used, the duration of treatment and follow-up, and the use of immunosuppressant. Five of the studies included patients treated with IFN alone (n = 48), four studies included IFN-RIB therapy (n = 33), and three studies included PEG-RIB therapy (n = 59). Immunosuppressive therapy at the beginning of antiviral therapy included cyclosporine A (CsA), tacrolimus (Tac)/FK506, azathioprine (Aza), corticosteroids (CS), and mycophenolatemophetil (MMF).

**Table 2:** Treatment schedules of IFN-based for HCV infection post-RT.

Authors	Interval from RT to treatment (months)	Treatment protocol	Duration of treatment (months)	Duration of follow-up (months)	Immunosuppression (name ; n/T)
Yasumura T	97.8±55.4	IFN 6 MU TIW	7.0±0.9	47.2±23.2	CsA 1/6;MZR 1/6;Prelon1/6;
et al [62]					
Izopet J	51.8±51.4	IFN 3MU TIW	4.7±1.2	12	CsA; Ste; AZ; MP;
et al [61]					
Durlik M.	60(60–180)	IFN 3MU TIW	6.2±2.2	6.7±1.5	Pred; CsA; AZ; MMF;
et al [63]					
Hanafusa T	NA	IFN 9MU TIW	6	24	Ste 3/10; OKT3 1/10;
et al [64]					
Tokumoto T	44.4±23.1	IFN 10MU TIW	6	20.8±3.7	MP; CsA; AZ; OKT3; DSG;
et al [65]					
Baid S	39.2±40.6	IFN 3MU TIW	18.3±14.8	23.7±18.4	Pred; AZ; CsA; Medrol;
et al [66]		RIB 200–800 mg/d			Tac; MMF;
Tang S	5.3±3.4	IFN 3MU TIW	6∼12	27.3±11.8	CsA;
et al [67]		RIB 400–1200 mg/d			
Shu K.H	32.4	IFN 1MU TIW	12	11.1±3.9	CsA; Tac; MMF; Medrol;
et al [68]		RIB 400–600 mg/d			
Sharma R	14.5±7.6	IFN 3MU TIW	12.4±5.5	NA	CsA; Pred;
et al [69]		RIB 600–800 mg/d			
Pageaux G.P	198.9±101.1	PEG 180 ug QW	6∼12	36(18–54)	Tac 2/8; MMF 2/8; Aza 3/8;
et al [70]		RIB 0–400 mg/d			CsA 4/8; Ste 8/8;
Aljumah A.A	66.3±45.7	PEG 80–180 µg QW	12	NA	Pre 19/19; MMF 15/19;
et al [71]		RIB 400–1200 mg/d			CsA 8/19; Tac 9/19;Siro1/19;
Sanai F.M	86.4±50.4	PEG 135–180 µg QW	12	6∼12	Tac 65.6%; Cy 28.1%;
et al [72]		RIB 200–1200 mg/d			MMF 87.5%;

AZ: azathioprine; CsA: cyclosporine A; CS: corticosteroids; DSG: deoxyspergualin; IFN: interferon; Medrol: Methylprednisolone; MMF: mycophenolate; MP: methylprednisolone; MZR: mizoribine; MU: million units; Tac: Tacrolimus; Siro: sirolimus; Pred: prednisone; Prelon: Prednisolone; RT: Renal Transplant; Ste: steroid; TIW: three times per week;

In three papers (Yasumura T et al/Hanafusa T et al/Tokumoto T et al) IFN was given on a daily dose for the first two weeks only;

In paper Pageaux G.P et al, PEG was given in three patients at 1.5 ug/kg/week,andone patient at 50 ug QW in paper Sharma R et al;

In two papers (Baid S et al/Tang S et al) the follow-up time calculated from the initiation of antiviral treatment.

In Table 3, the outcomes of IFN-based therapy of each study are shown, recorded as the virological and biochemical responses at the end of treatment and follow up at least six months. The overall SVR rate in PEG-based and standard IFN-based therapy was 40.6% (24/59) and 20.9% (17/81), respectively. Ten patients out of 140 experienced graft rejection and 31 patients out of 140 discontinued treatments because of side-effects such as graft-dysfunction, depression, Flu-like symptoms, anemia, and leucopenia. That is to say, the overall graft rejection rate and drop-out rate was 7% (10/140) and 22% (31/140), respectively.

**Table 3:** Outcome of studies of IFN-based therapy for HCV infection post-RT.

Authors	ETBR	ETVR	EFBR	SVR	Rejection rate	Discontinuing	Compliance(A/B/C; n/T)	Side-effect
Yasumura T	100%(6/6)	33.3%(2/6)	50%(3/6)	33.3%(2/6)	16.6%(1/6)	0	A(6/6)	Graft dysfunction (n = 1);
et al [62]								
Izopet J	80%(12/15)	33.3%(5/15)	27%(4/15)	0	0	46.7%(7/15)	A(8/15);C(7/15)	Graft dysfunction (n = 5); backache; fatigue;
et al [61]								anorexia; weight loss; alopecia etc;
Durlik M.	27.2%(3/11)	0	18.2%(2/11)	0	9.0%(1/11)	0	A(11/11)	Graft dysfunction (n = 2);
et al [63]								
Hanafusa T	30%(3/10)	20%(2/10)	20%(2/10)	10%(1/10)	40%(4/10)	50%(5/10)	A(5/10);C(5/10)	Graft dysfunction (n = 4);
et al [64]								
Tokumoto T	100%(6/6)	50%(3/6)	100%(6/6)	50%(3/6)	16.6%%(1/6)	33.3%(2/6)	A(4/6);C(2/6)	Graft dysfunction (n = 2);
et al [65]								
Baid S	75%(9/12)	33%(4/12)	25%(3/12)	33%(4/12)	16.6%(2/12)	16.6(2/12)	A(4/4);C(6RIB+2/12)	Graft dysfunction (n = 2) thrombocytopenia;
et al [66]								Flu-like syndromes; leucopenia; depression;
Tang S	75%(3/4)	75%(3/4)	50%(2/4)	50%(2/4)	0	0	A(3/4);B(1RIB/4)	0
et al [67]								
Shu K.H	91%(10/11)	64%(7/11)	27%(3/11)	27%(3/11)	0	27%(3/11)	A(8/11);C(3/11)	Graft dysfunction(n = 1) ;Flu-like syndromes;
et al [68]								urosepsis;depression;
Sharma R	33.3(2/6)	66.7%(4/6)	33.3%(2/6)	33.3%(2/6)	0	33.3%(2/6)	A(4/6);C(2IFN/6)	Graft dysfunction (n = 4);
et al [69]								Low platelets; anemia;
Pageaux G.P	100%(8/8)	75%(6/8)	100%(4/4)	50%(4/8)	0	62.5% (5/8)	A(2/8);C(5IFN+1RIB/8)	Graft dysfunction (n = 1);
et al [70]								depression; anemia; papillary oedema;
Aljumah A.A	79%(15/19)	47%(9/19)	79%(15/19)	42%(8/19)	5.3%(1/19)	0	A(19/19)	Graft dysfunction (n = 3)
et al [71]								
Sanai F.M	NA	47%(9/19)	NA	37.5%(12/32)	0	15.6%(5/32)	A?;B(25RIB+11PEG/32)	Graft dysfunction (n = 2); anemia;
et al [72]							C(5/32)	Flu-like syndrome; depression etc;

ETBR: end-of-treatment biochemical response; ETVR: end-of-treatment virological response; EFBR: biochemical response of follow-up at least 6 months; SVR: sustained virological response; Compliance (A/B/C): full duration, target dosages/full duration, reduced dosages/premature discontinuation.

### Data analysis

**Figure 2:**
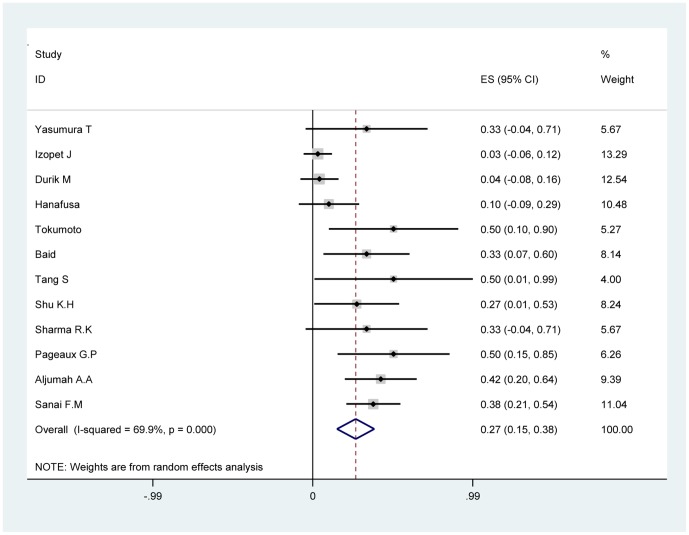
Forest map of summary estimate for SVR rate.

**Figure 3:**
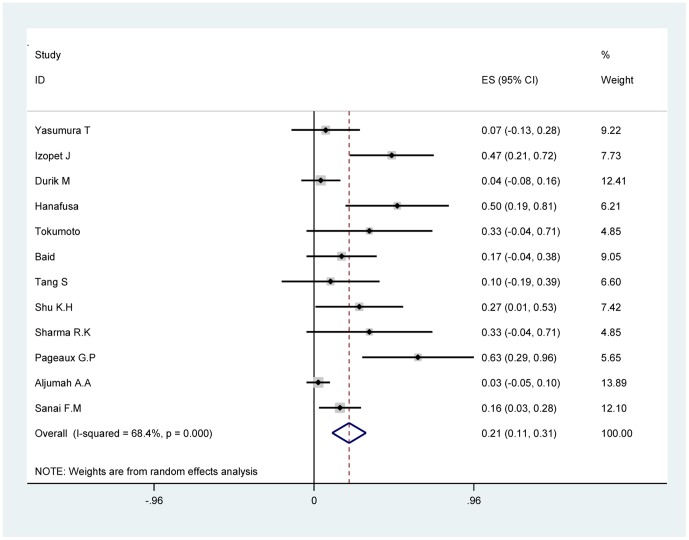
Forest map of summary estimate for Drop-out rate.

The quantitative pooled summary estimates for SVR and drop-out rate are shown in Table 4 and Table 5, respectively. The summary estimate for SVR rate and drop-out rate was 26.6% (95% CI: 15.0–38.1%) and 21.1% (95% CI: 10.9–31.2%), respectively. The heterogeneity Q-score was 36.53 and 34.85 for the SVR rate and drop-out rate, respectively. The I^2^ value was 69.9% and 68.4%for the SVR rate and drop-out rate respectively. The p-value was >0.10 for our test of study homogeneity, suggesting that the studies included were heterogeneous with respect to the outcome end-points. The summary estimate for ETBR rate, EFBR rate and ETVR rate was 63.6% (95% CI: 44–79.5%), 37.8% (95% CI: 24.9–52.5%) and 42.7% (95% CI: 27.7–57.6%), respectively. The summary estimate of graft rejection rate was 4% (95% CI: 0.8–7.1%). The forest map of SVR rate and drop-out rate are shown in Figure 2 and Figure 3 respectively.

**Table 4:** Summary estimates (with 95%CI) for SVR rate.

Author	SVR rate	[95% Conf. Interval]			Weight (%)
Yasumura T.et al [62]	0.333	−0.044	to	0.71	5.67
Izopet J.et al [61]	0.031	−0.057	to	0.119	13.29
Durik M.et al [63]	0.041	−0.076	to	0.158	12.54
Hanafusa T.et al [64]	0.1	−0.086	to	0.286	10.48
Tokumoto T.et al [65]	0.5	0.1	to	0.9	5.27
Baid S.et al [66]	0.333	0.066	to	0.6	8.14
Tang S.et al [67]	0.5	0.01	to	0.99	4
Shu K.H.et al [68]	0.272	0.009	to	0.535	8.24
Sharma R.K.et al [69]	0.333	−0.044	to	0.71	5.67
Pageaux G.P.et al [70]	0.5	0.154	to	0.846	6.26
Aljumah A.A.et al [71]	0.421	0.199	to	0.643	9.39
Sanai F.M.et al [72]	0.375	0.207	to	0.543	11.04
D+L pooled	0.266	0.15	to	0.381	100
Heterogeneity Q (p value)	36.53(0.000)				

**Table 5:** Summary estimates (with 95%CI) for Drop-out rate.

Author	Drop-out rate	[95% Conf. Interval]			Weight (%)
Yasumura T.et al [62]	0.07	−0.134	to	0.28	9.22
Izopet J.et al [61]	0.467	0.215	to	0.719	7.73
Durik M.et al [63]	0.041	−0.076	to	0.158	12.41
Hanafusa T.et al [64]	0.5	0.19	to	0.81	6.21
Tokumoto T.et al [65]	0.333	−0.044	to	0.71	4.85
Baid S.et al [66]	0.166	−0.045	to	0.377	9.05
Tang S.et al [67]	0.1	−0.194	to	0.394	6.6
Shu K.H.et al [68]	0.272	0.009	to	0.535	7.42
Sharma R.K.et al [69]	0.333	−0.044	to	0.71	4.85
Pageaux G.P.et al [70]	0.625	0.29	to	0.96	5.65
Aljumah A.A.et al [71]	0.025	−0.045	to	0.095	13.89
Sanai F.M.et al [72]	0.156	0.03	to	0.282	12.1
D+L pooled	0.211	0.109	to	0.312	100
Heterogeneity Q (p value)	34.85(0.000)				

### Sensitivity and heterogeneity analysis

The summary estimate for SVR rate in patients treated with IFN alone was 9.6% (95% CI: −0.9–20.2%), 32.8% (95% CI: 17.0–48.7%) in patients treated with IFN-RIB, and 40.6% (95% CI: 28.1–53.1%) in patients receiving PEG-RIB. The summary estimate for SVR rate in studies from Asian countries was 31.7% (95% CI: 20.5–43%); within the subgroup of cohort trials, the summary estimate for SVR rate was 26.3% (95% CI: 14.2–38.3%).

The summary estimate for drop-out rate in patients treated with IFN alone was 25.4% (95% CI: 5.0–45.7%), 24.4% (95% CI: 8.6–40.2%) in patients treated with IFN-RIB, and 20.1% (95% CI: −1.5–41.6%) in patients receiving PEG-RIB. In Asian countries, the summary estimate for drop-out rate was 16% (95% CI: 4.7–27.4%); within the subgroup of cohort studies, the summary estimate for drop-out rate was 20.5% (95% CI: 10.1–30.9%).

Graft dysfunction occurred in approximately one-fifth of RT recipients (27/140; 19.2%) who received IFN-based therapy for HCV infection. Although 13 patients who reported graft dysfunction were able to complete their treatment. A total of 31 patients discontinued treatment as a result of side-effects, including 14 patients cessation from treatment because of graft dysfunction. Thus, graft dysfunction was the most frequent side-effect of requiring discontinuation from treatment (14/31, 45%).Of the 12 reports included in our meta-analysis, only 3 used PEG-based therapies, and no studies included a control group. Thus, we were unable to conduct a subgroup analysis of IFN and PEG to calculate pooled odds ratios or mean differences in comparison.

As shown in Table S1 and Table S2, meta-regression analysis reported the variance between studies decreased from 0.0241 to 0 in SVR logit rate, suggesting the covariates included in the studies contribute to heterogeneity of the studies. The variance in drop-out rate logit rate between studies changed from 0.0179 to 0.04422 in meta-regression analysis, suggesting that covariates did not contribute to the heterogeneity. The sensitivity analyses by the random model yielded similar results to the fixed-effects model (Figures S1, S2, S3, S4).

### Publication bias

**Figure 4:**
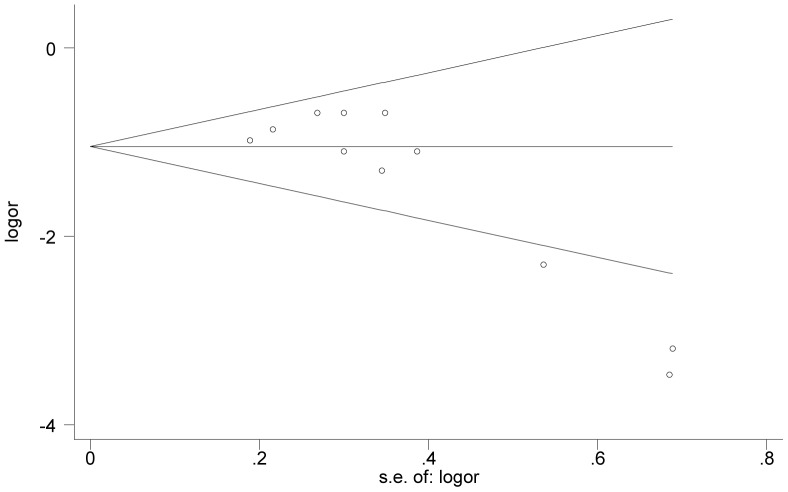
Funnel plot of precision by SVR logit rate.

**Figure 5:**
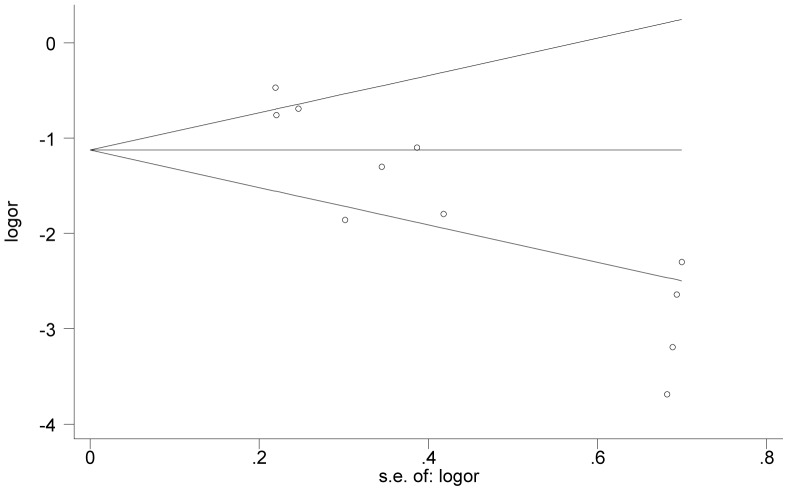
Funnel plot of precision by Drop-out logit rate.

The Egger and Begg tests for publication bias showed that the risk for missing trials was acceptably low. The funnel plots analyzing publication bias for SVR logit rate and Drop-out logit rate are shown in Figure 4 and Figure 5, respectively. The primary publication bias in our study is a preference for small cohort studies, with few large clinical trials.

## Discussion

IFN-based combination treatment of HCV infection in the immunocompetent, non-transplant population has been well-studied with large, randomized controlled clinical trials. Meta-analyses of these trials have demonstrated a SVR rate of approximately 41% in IFN-RIB and 55% in PEG-RIB [73], [74]. However, in post-RT patients with HCV infection, our present meta-analysis illustrates the limited efficacy, with a SVR rate of 32.8% in IFN-RIB and 40.6% in PEG-RIB, indicating reduced efficacy. Thus, the overall therapeutic advantage of IFN-RIB or PEG-RIB observed in non-transplant chronic HCV infection seemed to be attenuated post RT. Besides, IFN-based combination therapy is more efficient than IFN monotherapy, with at least a two-fold increase in SVR. Moreover, PEG-RIB has a higher SVR than IFN-RIB. The overall SVR in PEG-based therapy is much higher than that of standard IFN-based therapy. This result indicates that PEG-based therapy is a more effective approach for treating HCV infection post-RT than standard IFN-based therapy.

This systematic review showed that the rate of graft rejection was small, with a summary estimate of 4% (0.8–7.1%). At present, the exact mechanism of graft rejection triggered by IFN in RT recipients is not clear. IFN is a known to be a strong immune modulator, thus, rejection post-RT may involve an immune response. Potential pathways include increased cell surface expression of HLA antigens and induction of cytokines with subsequent stimulation of antibody production by B-cells [75]. It is interesting that the risk of rejection due to IFN is probably lower in liver than in RT recipients, this indicates that the liver being considered as more resistant to rejection than the kidney [76]. In addition, Baid et al noted the risk for acute rejection is higher during the first year after transplantation surgery [66]. Thus, it is strongly recommended to wait at least one year after the surgery to initiate antiviral therapy. Furthermore, antiviral treatment may yield a more effective response if stable renal function and no acute rejection occur during the first year after transplantation [21].

Currently, the limited available data suggests that amantadine monotherapy is safe and tolerated but has limited efficacy in managing HCV infection [77], [78]. Analogously, ribavirin monotherapy appeared to have some biochemical efficacy, but there is no consensus on its affects on liver histology. Furthermore, ribavirin can induce Hemolytic anemia, a serious side effect, though it has been reported to improve the level of proteinuria in HCV-related de novo glomerulopathy. As these data show, the existing alternatives to monotherapies are not clinically effective. Thus it is important to either improve IFN-based therapies or develop novel therapeutic approaches to manage HCV infection post-RT. In recent years, novel protease and polymerase inhibitor agents (e.g. Telaprevir and Boceprevir) were licensed to treat HCV infection. However, they have never been studied in the post-RT population and the newer second generation protease inhibitors as well as the NS5b polymerase inhibitors have likewise not been used, or licensed for use in this important population. These agents may provide additional candidates for combination therapy with PEG-RIB to improve patient outcome.

The ultimate goal of IFN-based treatment of HCV infection post-RT is the eradication of the infection and prevention of HCV-related liver damage. However, as our meta-analysis indicates, IFN therapy has limited efficacy and may induce graft rejection. Therefore, not all RT recipients who are HCV seropositive should receive IFN-based antiviral therapy. The guideline of Kidney Disease Improving Global Outcome (KDIGO) suggests that IFN therapy should limited to cases of recurrent or progressive HCV-related Glomerulopathy in the transplant kidney, and advanced liver diseases such as liver fibrosis or fibrosing cholestatic hepatitis [29], [79], [80].The strategy of using of IFN therapy to treat HCV infection after RT is based largely on the positive results of this approach in non-transplant settings. However, unlike in non-transplant setting, there are no large, controlled clinical trials to test the efficacy of IFN therapy in post-RT patients. Instead, most of the published reports on IFN therapy post-RT describe small cohort studies. Without detailed clinical trials, it is difficult to predict the efficacy and tolerability of IFN therapy in post-RT patients. The present meta-analysis of 12 clinical trials is the first study, to our knowledge, to pool the results of multiple studies testing the efficacy of IFN-based antiviral therapy for treating HCV infection post-RT.

Compared to the previous meta-analysis of IFN-based therapy post-RT [18], our analysis employed more strict inclusion and exclusion criteria, more accurate data extraction, and incorporated the biochemical response rate and graft rejection rate. Furthermore, earlier studies (included in the prior meta-analysis) used IFN dosages that are unlikely to produce optimal SVR. In addition, some of these early studies did not describe the method for diagnosing graft rejection, which can potentially cause over-diagnosis of the condition [81]. Due to these factors, the previous meta-analysis may have overestimated the drop-out rate while underestimating the SVR. As a result, our updated meta-analysis may provide a more reliable conclusion regarding the efficacy of IFN therapy in the post-RT setting. Moreover, our meta-analysis included reports of the PEG-based therapies, which have a more beneficial effect on virological and biochemical response than standard IFN-therapies. This finding could have a significant impact on future treatment strategies for HCV patients, as it suggests that PEG-based therapy can be employed to improve the limited efficacy of IFN therapy.

The results of this meta-analysis should facilitate treatment decisions for post-RT patients with HCV infection. Emerging evidence suggests that HCV-related therapy should be performed in patients prior to renal transplantation because when HCV RNA clearance occurred, they experienced no relapse after transplantation despite chronic immunosuppressive treatment [53]. The results of our meta-analysis should be interpreted in the context of the limitations of the included studies. For example, our analysis consisted of eleven small cohort studies and only one controlled clinical trial, without any large, randomized, controlled clinical trials. Given the stringency of our eligibility criteria, this publication bias likely reflects the need for more comprehensive research on the efficacy of IFN in post-RT patients. Another limitation of the included studies was the lack of a control group (e.g. placebo treated patients). As a result, we were unable to calculate pooled odds ratios or mean differences in comparison to placebo or other therapies. Therefore, it is difficult to provide an accurate estimate of the efficacy and tolerability of IFN treatment in patients with HCV infection post-RT. Additionally, our analysis does not include histological data from the end of treatment, time-points beyond six months of follow up, or patients with end-stage renal disease.

In conclusion, the present review and meta-analysis demonstrates the limited safety and efficacy of IFN-based antiviral therapy for HCV infection post-RT. The therapeutic advantage of IFN-RIB or PEG-RIB therapy observed in non-transplant chronic HCV infection appears to be attenuated post RT.However, PEG-RIB demonstrates greater efficacy on virological and biochemical response compared to IFN-RIB in patients with HCV infection post-RT. We believe this meta-analysis further advances the field of transplant hepatology by clarifying the benefits and risks of IFN-based antiviral therapy post-RT. In particular, our study suggests that the limited benefits of IFN-based therapy post RT need to be weighed against the risk of allograft rejection. Future research is required to develop novel strategies to improve therapeutic efficacy and tolerability, and reduce the liver-related morbidity and mortality in this important patient population.

## Supporting Information

Checklist S1 **The PRISMA checklist.**
(DOC)

Figure S1 **Sensitivity analysis by fixed-effects model of SVR logit rate.**
(DOC)

Figure S2 **Sensitivity analysis by random-effects model of SVR logit rate.**
(DOC)

Figure S3 **Sensitivity analysis by fixed-effects model of Drop-out logit rate.**
(DOC)

Figure S4 **Sensitivity analysis by random-effects model of Drop-out logit rate.**
(DOC)

Table S1 **Meta-regression analysis (dependent variable: SVR logit rate).**
(DOC)

Table S2 **Meta-regression analysis (dependent variable: Drop-out logit rate).**
(DOC)
