# Supplementary material for: Interferon-Based Anti-Viral Therapy for Hepatitis C Virus Infection after Renal Transplantation: An Updated Meta-Analysis
Source: PLoS One. 2014 Apr 3;9(4):e90611. doi: 10.1371/journal.pone.0090611 (PMC3974660; doi:10.1371/journal.pone.0090611)
Supplement: Figure S4 — Sensitivity analysis by random-effects model of Drop-out logit rate. (DOC) [file pone.0090611.s005.doc]

**Figure S4: Sensitivity analysis by random-effects model of Drop-out logit rate.**
