# Supplementary material for: Interferon-Based Anti-Viral Therapy for Hepatitis C Virus Infection after Renal Transplantation: An Updated Meta-Analysis
Source: PLoS One. 2014 Apr 3;9(4):e90611. doi: 10.1371/journal.pone.0090611 (PMC3974660; doi:10.1371/journal.pone.0090611)
Supplement: Table S1 — Meta-regression analysis (dependent variable: SVR logit rate). (DOC) [file pone.0090611.s006.doc]

**Table S1: Meta-regression analysis (dependent variable: SVR logit rate).**

| Covariates Coef. Std. Err t P>|t| [95% Conf. Interval ] | | | | | | |
| --- | --- | --- | --- | --- | --- | --- |
|
| Age | 0.0236649 | 0.0489838 | 0.48 | 0.713 | -0.5987338 | 0.646036 |
| Male percentage | 0.042788 | 0.0107781 | 3.92 | 0.159 | -0.0946704 | 0.179228 |
| Reference year | 0. 44163 | 0.0473741 | 3.89 | 0.16 | -0.4175288 | 0.786362 |
| Cirrhosis rate | 0.3252092 | 0.2177103 | 1.49 | 0.376 | -2.441062 | 3.09148 |
| Donor source | 0.456408 | 0.518117 | 0.88 | 0.54 | -6.126893 | 7.039709 |
| RT-time pre-IFN | -0.0071546 | 0.0048644 | -1.47 | 0.38 | -0.0689622 | 0.054653 |
| IFN dose | 0.3381584 | 0.1034967 | 3.27 | 0.189 | -0.9768914 | 1.653208 |
| Treatment duration | 0.1080412 | 0.0556427 | 1.94 | 0.303 | -0.598966 | 0.815048 |
