# Supplementary material for: Interferon-Based Anti-Viral Therapy for Hepatitis C Virus Infection after Renal Transplantation: An Updated Meta-Analysis
Source: PLoS One. 2014 Apr 3;9(4):e90611. doi: 10.1371/journal.pone.0090611 (PMC3974660; doi:10.1371/journal.pone.0090611)
Supplement: Table S2 — Meta-regression analysis (dependent variable: Drop-out logit rate). (DOC) [file pone.0090611.s007.doc]

**Table S2: Meta-regression analysis (dependent variable: Drop-out logit rate**).

| Covariates Coef. Std. Err t P > |t| [95% Conf. Interval] | | | | | | |
| --- | --- | --- | --- | --- | --- | --- |
| Age | 0.1713865 | 0.062717 | 2.73 | 0.223 | -0.6255078 | 0.9682807 |
| Male percentage | -0.0133702 | 0.015364 | -0.87 | 0.544 | -0.2085848 | 0.1818444 |
| Reference year | -0.0256463 | 0.056005 | -0.46 | 0.727 | -0.737258 | 0.6859654 |
| Cirrhosis rate | 0.7462585 | 0.267115 | 2.79 | 0.219 | -2.74776 | 4.140277 |
| Donor source | 0.6065419 | 0.707204 | 0.86 | 0.549 | -8.379341 | 9.592425 |
| RT time pre-IFN | -0.0025255 | 0.006604 | -0.38 | 0.767 | -0.0864406 | 0.0813896 |
| IFN dose | 0.2150097 | 0.13186 | 1.63 | 0.35 | -1.460429 | 1.890449 |
| Treatment duration | 0.0506355 | 0.079433 | 0.64 | 0.639 | -0.9586559 | 1.059927 |
